# Differential proteomic analysis of grapevine leaves by iTRAQ reveals responses to heat stress and subsequent recovery

**DOI:** 10.1186/1471-2229-14-110

**Published:** 2014-04-28

**Authors:** Guo-Tian Liu, Ling Ma, Wei Duan, Bai-Chen Wang, Ji-Hu Li, Hong-Guo Xu, Xue-Qing Yan, Bo-Fang Yan, Shao-Hua Li, Li-Jun Wang

**Affiliations:** 1 Key laboratory of Plant Resources and Beijing Key Laboratory of Grape Science and Enology, Institute of Botany, Chinese Academy of Sciences, Beijing 100093, P. R., China; 2 University of China Academy of Sciences, Beijing 100049, P. R., China; 3 Key Laboratory of Photobiology, Institute of Botany, Chinese Academy of Sciences, Beijing 100093, P. R., China; 4 Beijing Computing Center, Beijing 100094, P. R. China; 5 Key laboratory of Plant Germplasm Enhancement and Specialty Agriculture, Wuhan Botany Garden, Chinese Academy of Sciences, Wuhan 430074, P. R., China

**Keywords:** Cabernet sauvignon, Heat stress, iTRAQ, Photosynthesis, Proteomics, Recovery

## Abstract

**Background:**

High temperature is a major environmental factor limiting grape yield and affecting berry quality. Thermotolerance includes the direct response to heat stress and the ability to recover from heat stress. To better understand the mechanism of the thermotolerance of *Vitis*, we combined a physiological analysis with iTRAQ-based proteomics of *Vitis vinifera* cv Cabernet Sauvignon, subjected to 43°C for 6 h, and then followed by recovery at 25/18°C.

**Results:**

High temperature increased the concentrations of TBARS and inhibited electronic transport in photosynthesis apparatus, indicating that grape leaves were damaged by heat stress. However, these physiological changes rapidly returned to control levels during the subsequent recovery phase from heat stress. One hundred and seventy-four proteins were differentially expressed under heat stress and/or during the recovery phase, in comparison to unstressed controls, respectively. Stress and recovery conditions shared 42 proteins, while 113 and 103 proteins were respectively identified under heat stress and recovery conditions alone. Based on MapMan ontology, functional categories for these dysregulated proteins included mainly photosynthesis (about 20%), proteins (13%), and stress (8%). The subcellular localization using TargetP showed most proteins were located in the chloroplasts (34%), secretory pathways (8%) and mitochondrion (3%).

**Conclusion:**

On the basis of these findings, we proposed that some proteins related to electron transport chain of photosynthesis, antioxidant enzymes, HSPs and other stress response proteins, and glycolysis may play key roles in enhancing grapevine adaptation to and recovery capacity from heat stress. These results provide a better understanding of the proteins involved in, and mechanisms of thermotolerance in grapevines.

## Background

Temperature is one of the pivotal factors influencing plant growth and development. Both yield and quality are reduced when the temperature is above or below optimal levels [1]. The IPCC (Intergovernmental Panel on Climate Change) forecasts that the extreme annual daily maximum temperature (i.e., return value) will likely increase by about 1-3°C by mid-twenty-first century and by about 2-5°C by the late twenty-first centry (http://www.ipcc.ch), and direct grape yield losses in the range of 2.5-16% for every 1°C increase in seasonal temperatures have been observed [2]. Therefore, a better understanding of the mechanisms involved in thermotolerance would be greatly significant and would lay the theoretical foundation for formulating the strategies of adaptation to high temperatures.

Direct injuries associated with high temperatures include protein denaturation, aggregation, and increased fluidity of membrane lipids. Indirect or slower heat injuries include inactivation of enzymes in chloroplasts and mitochondria, inhibition of protein synthesis, protein degradation and loss of membrane integrity [3,4]. Photosynthesis is a very sensitive process to heat stress. The inhibition of photosystem (PS) II leads to a change in variable chlorophyll *a* fluorescence, and *in vivo* chlorophyll may be used to detect changes in the photosynthetic apparatus, for example, with an O-J-I-P test [5,6]. Heat stress also affects the organization of microtubules by splitting and/or elongating the spindles, forming microtubule asters in mitotic cells, and elongating the phragmoplast microtubules [7]. These injuries eventually lead to starvation, inhibition of growth, reduced ion flux, and the production of toxic compounds and reactive oxygen species (ROS) [3,8]. To counter the effects of heat stress on cellular metabolism, plants and other organisms respond to temperature changes by reprogramming their transcriptome, proteome, metabolome and lipidome; that is, by altering their composition of certain transcripts, proteins, metabolites and lipids. Such changes are aimed at establishing a new steady-state balance of metabolic processes that can enable the organism to function, survive and even reproduce at a higher temperature [4]. In general, most of the previous studies about heat stress focused on physiological or transcriptomic approaches. As protein metabolic processes, including synthesis and degradation, are most sensitive to heat stress, proteomics research on heat stress could have a large impact on the understanding of its consequences.

Proteomics became popular in the 1990s and has greatly evolved to a mature stage today. The most frequently used proteomic technique is the two-dimensional (D) gel technique, where differentially expressed spots are excised and analyzed by mass spectrometry (MS). Proteomic responses to heat stress have been widely studied in many species, including rice [9,10], wheat [11,12], barley [13], *Populus euphratica*[14], Norway spruce [15], bitter gourd [16]. However, not all types of proteins are amenable to gel-based electrophoresis and the dynamic range is somewhat limited [17]. Additionally, the co-migration and partial co-migration of proteins can compromise the accuracy of the quantification, and interfere with protein identification [17,18]. In recent years, a new technique termed iTRAQ (isobaric tags for relative and absolute quantitation) has been applied for proteomic quantitation. iTRAQ labeling overcomes some of the limitations of 2D-gel-based techniques, and also improves the throughput of proteomic studies. This technique has a high degree of sensitivity, and the amine specific isobaric reagents of iTRAQ allow the identification and quantitation of up to eight different samples simultaneously [17,19,20].

Grapevines are widely cultivated fruit vines around the world, and are mainly used for juice, liquor and wine production [21]. Heat stress is known to retard the growth and development of grapes, resulting in the decline of the yield and quality of the berry [22]. Similar to other plants, the previous studies on the response of grapevines to high temperatures have mainly focused on physiological changes including photosynthesis, respiration, cell membrane stability, hormone changes and antioxidant systems [22-29]. However, the underlying mechanisms of heat stress are still unclear. Transcriptomic analysis of grape (*Vitis vinifera* L.) leaves was conducted using the Affymetrix Grape Genome oligonucleotide microarray (15,700 transcripts) under heat stress and subsequently recovery [29]. The effect of heat stress and recovery on grape appears to be associated with multiple processes and mechanisms including stress-related genes, transcription factors, and metabolism [29]. However, the transcription patterns are not always directly concomitant with protein expression levels [30], and there are currently no reports on proteomic analyses in grapevines under heat stress. There have been, however, several reports of proteomic analyses of grapes (fruit). In order to understand the berry development and ripening process, Martı’nez-Esteso *et al.* (2011) correlated the proteomic profiles with the biochemical and physiological change occurring in grapes. They identified and quantified 156 and 61 differentially expressed proteins in green and ripening phases, respectively, through a top-down proteomic approach based on difference gel electrophoresis (DIGE) followed by tandem mass spectrometry (MS/MS) [31]. Basha *et al.* used the 2D-PAGE to identify unique xylem sap proteins in *Vitis* species with Pierce’s disease (PD), a destructive bacterial disease of grapes caused by *Xylella fastidiosa*[32]. Martı’nez-Esteso *et al.* (2011) also identified 695 unique proteins in developing berries using the iTRAQ labeling technique, with quantification of 531 proteins [33]. Therefore, although there are many reports on the proteome of grapes, most have focused on fruit development [31,33-35] and fruit disease [36-40]. To the best of our knowledge, there are only a few grape proteomic studies which have addressed grapevine responses to abiotic stresses, including water or salt stress [41-43]. None of these studies have yet addressed heat stress of grape leaves. Moreover, although the responses of some plants to stress are generally well-studied, relatively few studies have focused on the mechanisms associated with recovery after stress [44-47]. This recovery process from heat stress in plants is very important to survival, and the degree of recovery from stress is a direct index of plant thermotolerance [44]. As, there are potentially differences between the recovery and the direct heat response mechanisms in plants [48], a proteomic evaluation and comparison of these processes is warranted.

In this study, we used the iTRAQ labeling technique to assess proteome changes in 'Cabernet sauvignon’ leaves of *V. vinifera* under heat stress and their subsequent recovery, in order to better understand the thermotolerance mechanism in grapevines.

## Results

### Thermostability of cell membranes in grapevine leaves under heat stress and subsequent recovery

**Figure 1:**
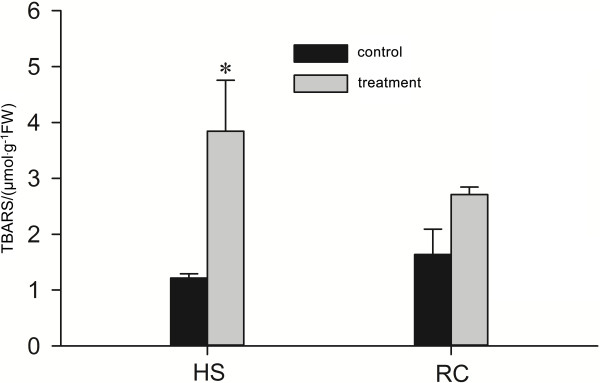
**TBARS in grape leaves under heat stress and subsequent recovery.** It is showed that heat treatment (43°C for 6 h) significantly increased the TBARS concentrations in grape leaves and after subsequent recovery, there was no difference in TBARS concentrations between heat-treated and control leaves. Each value represents the mean ± standard error of the mean (S.E.M.) of three replicates. The asterisks indicate the significance of differences between treatments and their corresponding controls (* *P* < 0.05).

The present study investigated changes in the cell membrane thermostability of 'Cabernet Sauvignon’ grapevine leaves under heat stress and subsequent recovery. We used the thiobarbituric acid reactive substances (TBARS) concentrations as an indicator of the peroxidation and destruction of lipids with subsequent membrane damage [9]. One-way ANOVA analysis showed that heat treatment (43°C for 6 h) significantly increased the TBARS concentrations in grape leaves (Figure 1), indicating the occurrence of damage to the cell membrane in the grapevine leaves under the heat treatment. After subsequent recovery, there was no difference in TBARS concentrations between heat-treated and control leaves (Figure 1).

### Changes in the electron transport chain of PSII under heat stress and subsequent recovery

**Figure 2:**
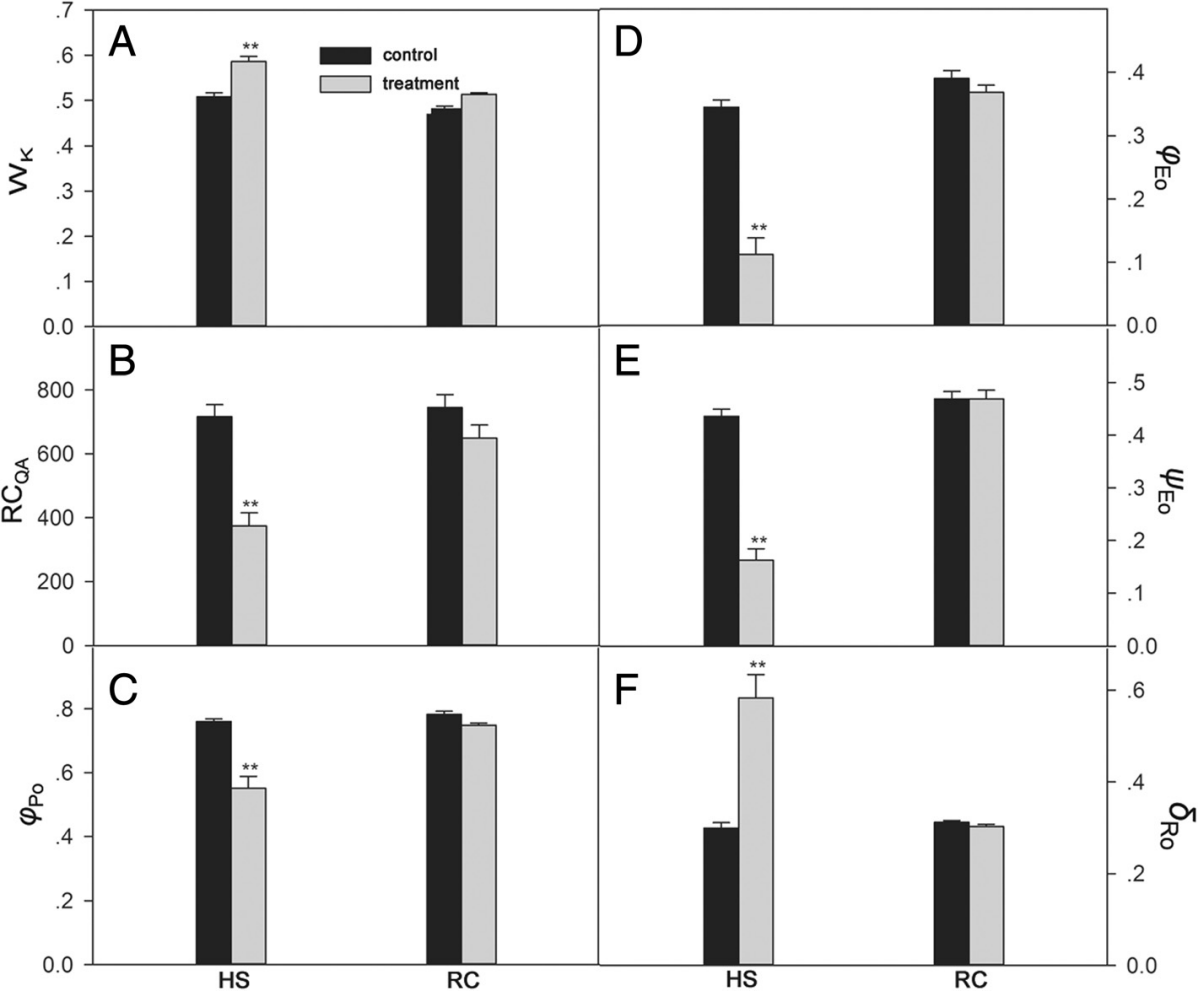
**Donor side (W**_**k**_**), reaction center (RC**_**QA**_**), acceptor side (*φ***_**Po**_**, *ψ***_**Eo**_**, *φ***_**Eo**_**) parameters of PSII and *δ***_**Ro **_**(the efficiency with an electron can move from plastoquinone (PQ) through PSI to the PSI end electron acceptor) in grape leaves under heat stress and subsequent recovery.** Each value represents the mean ± S.E. of five replicates. The asterisks indicate the significance of differences from their corresponding control (* *P* < 0.05, ** *P* < 0.01). The detailed meanings of W_k_, RC_QA_, *φ*_Po_, *ψ*_Eo_, *φ*_Eo_ and *δ*_Ro_ were shown in Additional file 7.

The O-J-I-P test was used to investigate changes in the electron transport chain of PSII. It has been shown that heat stress can induce a rapid rise in the O-J-I-P test. This phase, occurring at around 300 μs and labeled as K, is caused by an inhibition of the oxygen evolution complex (OEC). The amplitude of step K (W_k_) can therefore be used as a specific indicator of damage to the PSII donor site [49]. In addition, RC_QA_ indicates the density of the active section of Q_A_-reducing PSII reaction centers. In the present study, compared with the control (un-stressed conditions), heat stress resulted in an elevated W_K_ and a lowered RC_QA_ value (Figure 2A, B). After recovery, W_K_ declined and RC_QA_ ascended to the control levels. Figure 2C, D, E demonstrates the changes in maximum quantum yield for primary photochemistry (*φ*_Po_), the quantum yield for electron transport (*φ*_Eo_), the probability that a trapped excitation moves an electron into the electron transport chain beyond Q_A_^-^ (*ψ*_Eo_) in grape leaves during high temperature stress and recovery, respectively. *φ*_Po_, *φ*_Eo_, *ψ*_Eo_ decreased in grape leaves under heat stress, and went back to the control levels after recovery. *δ*_Ro_ signifies the redox state of photosystem I (PSI), i.e., the efficiency with which an electron transfers from plastoquinone (PQ) through PS I to reduce the PS I end electron acceptors. The *δ*_Ro_ value at 43°C rose significantly. However, these parameters returned to control levels after recovery (Figure 2C-F).

### Protein response to heat stress and/or recovery in grape leaves revealed by iTRAQ analysis

**Figure 3:**
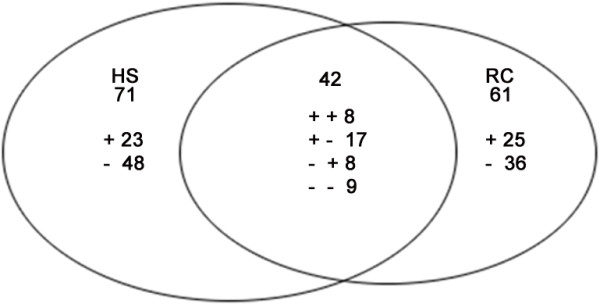
**Venn diagram of differentially expressed proteins that were up- or downregulated by heat stress or recovery.** The “ + “ and “- “indicate up- and downregulated proteins, respectively.

Two hundred and seventy-four proteins were quantified with at least one significant peptide sequence and 174 of these characterized proteins were differentially expressed, i.e. an expression ratio > 1.50 or < 0.67 [50-53] under heat stress or recovery compared to their corresponding controls. Heat stress and recovery affected protein expression levels in various ways. During heat stress, 48 proteins were upregulated, and 65 were downregulated, while 41 were upregulated and 62 were downregulated after recovery, compared to their corresponding control levels. There were 71 (23 up- and 48 downregulated) proteins and 53 (19 up- and 34 downregulated) proteins responding to only heat stress or recovery, respectively, while 42 proteins were differentially expressed in both heat stress and recovery. Among these 42 proteins, eight proteins were upregulated both under heat stress and recovery, while nine proteins showed an opposing trend under the two conditions. Seventeen proteins were upregulated under heat stress and downregulated during recovery, while eight proteins were downregulated under heat stress but upregulated during recovery. In addition, six upregulated proteins and two downregulated proteins were only identified under recovery from heat stress (Figure 3).

### Functional classification, subcellular localization and enrichment analysis of differentially expressed proteins under heat stress and subsequent recovery

**Figure 4:**
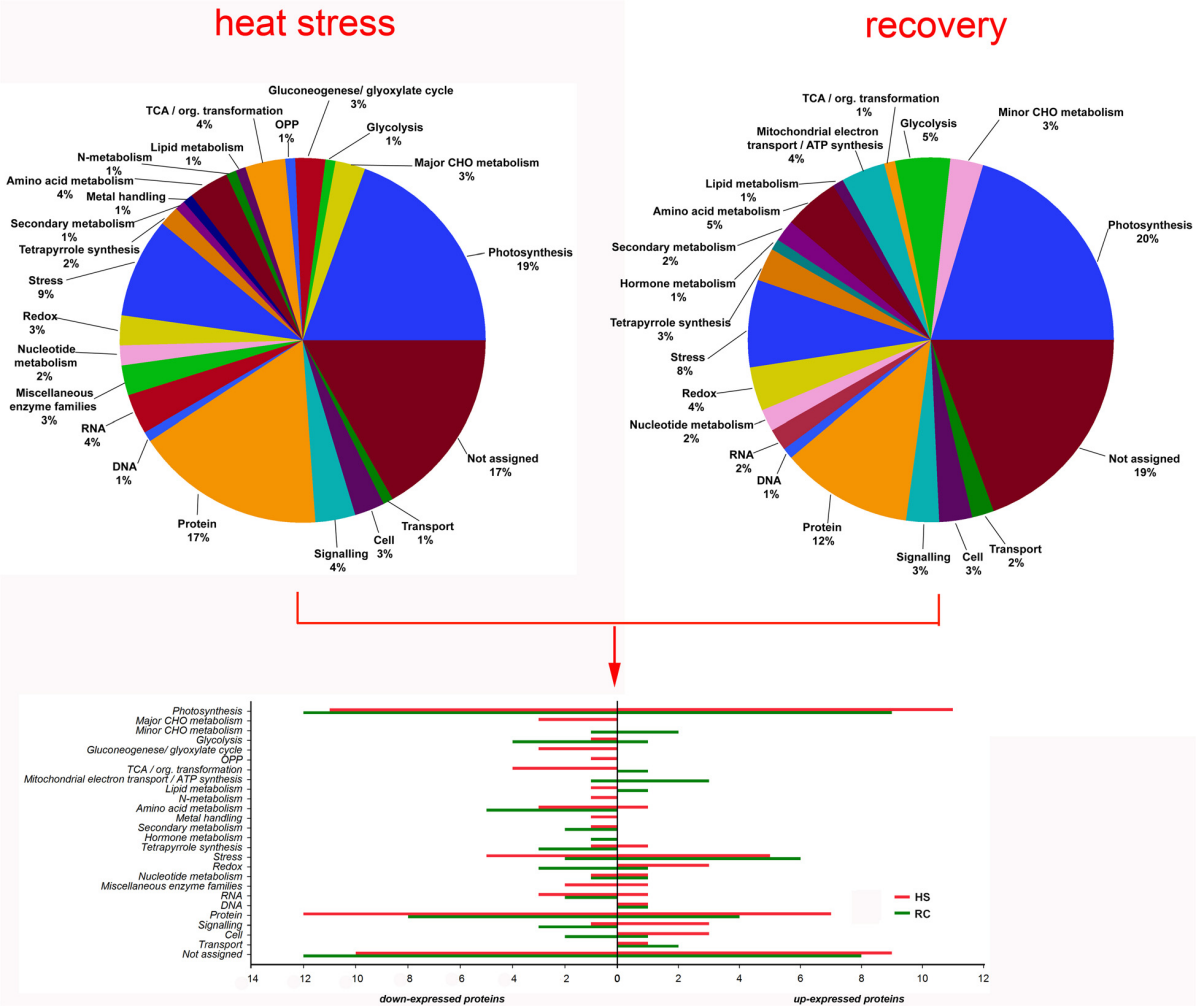
Functional characterization of heat stress and recovery–responsive proteins under heat stress and/or subsequent recovery.

**Figure 5:**
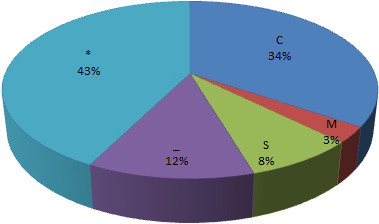
**Subcellular localization of the 174 differentially expressed proteins under heat stress and/or subsequent recovery.** C: Chloroplast, i.e. the sequence contains cTP, a chloroplast transit peptide; M: Mitochondrion, i.e. the sequence contains mTP, a mitochondrial targeting peptide; S: Secretory pathway, i.e. the sequence contains SP, a signal peptide; _: Any other location; *: “don't know”.

Among the 174 differentially expressed proteins, 127 were characterized as hypothetical or unknown proteins under the grape genomics information category in uniprot (http://www.uniprot.org/). To gain functional information about these proteins, BLASTP (http://www.ncbi.nlm.nih.gov/BLAST/) was used to search for homologous proteins against the NCBI non redundant (Nr) protein database. BLAST searching was able to align 117 of the unknown proteins (Additional file 1). Among these aligned proteins, 90.6% had an E-value of less than 1.0E-50 and showed very strong homology while the remaining 9.4% had an E-value of between 1.0E-10 and 1.0E-50. The identities distribution defined 27.4% of these aligned proteins as having a matched identity greater than 90%, 71.8% between 60% and 90% and only one protein (59.93%) lower than 60%. These results indicating that the unknown proteins might have similar function with the aligned proteins respectively. These differentially expressed proteins were classified into 26 functional categories according to MapMan ontology as shown in Figure 4 and Additional file 2. The main categories included photosynthesis, proteins and stress. In addition, enrichment analysis against agriGO (http://bioinfo.cau.edu.cn/agriGO/) showed that differentially expressed proteins were mainly enrich in response to abiotic stimulus (GO: 0009628), generation of precursor metabolites and energy (GO: 0006091) and photosynthesis (GO: 0015979) of biological process. Moreover, subcellular localization of the 174 characterized proteins showed that 60 proteins (34%) were located in chloroplast, five proteins (3%) were assigned to the mitochondria, 14 proteins (8%) belonged to secretory pathway, and 21 proteins (12%) were classified as belonging to other locations. Unfortunately, 74 of the differentially-expressed proteins had unknown locations (Figure 5). These results indicated that quite a lot of chloroplast proteins are related to thermotolerance of grapevine.

### Comparative analysis of common responsive proteins between heat stress and subsequent recovery

There were 17 proteins that were upregulated by heat stress, but were then downregulated after recovery (Additional file 3). Three of these proteins were categorized as being related to photosynthesis, including PSI reaction center subunit N (PsaN), ATP synthase subunit beta (fragment), and Rubisco large chain. Interestingly, PsaN was upregulated 28 fold by heat stress but then downregulated more than 5 fold after recovery, compared with their corresponding controls. In addition, two of the proteins were related to metabolism: one is acetoacetyl-CoA thiolase, which condenses two molecules of acetyl-CoA to give acetoacetyl-CoA, and this is the first enzymatic step in the biosynthesis of isoprenoids via mevalonate, the other is coproporphyrinogen-III oxidase (CPOX), a key enzyme in the biosynthetic pathway of chlorophyll. Universal stress protein (USP), a transcription factor in abiotic stress, and thylakoidal ascorbate peroxidase (APX), involved in H_2_O_2_ detoxification, were also induced by heat stress and decreased after subsequent recovery. Moreover, proteins related to protein metabolism included one chloroplastic large subunit ribosomal protein (L12-1) and one translation initiation factor (eIF3f). Peptidyl-prolyl cis-trans isomerase and two transporters, the nascent polypeptide associated complex alpha and the mitochondrial import inner membrane translocase subunit Tim9 were also affected. One14-3-3-like protein, associated with a DNA binding complex that binds to the G-box was also identified.

Only eight proteins were upregulated by both heat stress and subsequent recovery (Additional file 3). One PSII subunit R (PsbR), one PSI subunit H (PsaH) and a Rubisco small submit were induced after heat stress and recovery. Additionally, two ribosomal proteins (S21e, S9) were also identified. Moreover, heat shock protein (HSP) 26 in chloroplast was induced 3.4 and 2.0 fold respectively by heat stress and recovery. Nucleoside diphosphate kinase 1 (NDPK1), involved in purine metabolism, was also induced more than 10 fold under heat stress, and returned to almost the control level after recovery.

Eight proteins were downregulated by heat stress but upregulated after subsequent recovery (Additional file 3). Among the eight proteins, two of them are related to photosynthesis, PSI subunit l (PsaA), PSII protein D1 (PsbA). Biotin carboxylase subunit, a component of the acetyl coenzyme A complex was downregulated 0.46 fold by heat stress but upregulated 1.6 fold after subsequent recovery. In addition, two stress-related proteins of the HSP90 family (HSP90-5, HSP90-7) were also identified. The three remaining proteins in this group were not assigned.

Additional file 3 shows nine proteins that were downregulated both by heat stress and subsequent recovery. Light-harvesting chlorophyll-protein complex II subunit B1 (LHCB1.4) in photosynthesis and a magnesium-chelatase (MgCh) subunit ChlI-2 involved in chlorophyll biosynthesis were identified in this group. Cyanate hydratase which catalyzes the bicarbonate-dependent breakdown of cyanate to ammonia and bicarbonate in cyanogenic glycosides was also repressed both by heat stress and recovery. In addition, small subunit ribosomal protein SA and protein phosphatase 2C in protein metabolism was also repressed after heat stress and recovery.

### Analysis of proteins only responsive to heat stress

A total of 71 proteins showed a specific response to heat stress, with 23 upregulated proteins, and 48 downregulated proteins (Additional file 4). Five of the 23 upregulated proteins are related to photosynthesis, including PsaF, three ATP synthase subunits (γ, δ, b) involved in the photosystem electron-transfer reaction, and a fructose bisphosphate aldolase (FBA) involved in the Calvin cycle. Of note, the ATP synthase CF (0) b subunit was upregulated 8.4 fold by heat stress. Ribosomal protein S1 involved in protein synthesis was also upregulated by heat stress. HSP22, located in the endoplasmic reticulum, and HSP21, located in the chloroplast, were induced 3.0 and 5.5 fold, respectively, under heat stress. Cytoplasmic [Cu-Zn] superoxide dismutase (SOD), involved in redox, was also induced 5.0 fold under heat stress. In addition, 14-3-3-like protein was upregulated 1.8 fold by heat stress. Among the 48 downregulated proteins (Additional file 4), eight of them were involved in photosynthesis, including LHCB1.3, PsbP, and PsaL. Many other proteins were involved in a variety of metabolic mechanisms, including glucose-1-phosphate adenylyltransferase, two malate dehydrogenase enzymes (MDH), nitrite reductase 1 in N-metabolism and uracil phosphoribosyltransferase involved in nucleotide metabolism. There are also some carbohydrate metabolism-related proteins, such as UDP-glucose pyrophosphorylase, which catalyze the reversible reaction between glycose-1-phosphate and UDP-glycose, dihydrolipoyl dehydrogenase in the tricarboxylic acid cycle (TCA) and 6-phosphogluconate dehydrogenase in the oxidative pentose phosphate pathway (OPP). Three proteins were identified as being stress-related; including osmotin-like protein and HSP70. Two identified proteins, Beta-1-3 glucanase and alcohol dehydrogenase, were annotated to miscellaneous enzyme families. In addition, ten proteins were involved in protein metabolism, including mitochondrial-processing peptidase subunit α and β, in protein targeting; methionine sulfoxide reductase A, in posttranslational modification; protease Do-like 8, and proteasome subunit α type-5 in protein degradation and a 20 kDa chaperonin, involved in protein folding. There are also five proteins are not assigned.

### Analysis of proteins only responsive to recovery from heat stress

There were 25 proteins which were only upregulated after recovery from heat stress (Additional file 5). Four of these proteins are photosynthesis-related, including LHCB2.1, PsbS, PetB. Two upregulated stress proteins corresponded to the HSP70 family (HSP70-5, HSP70-11). HSP70-5 is located in the cytoplasm, while HSP70-11 is located in the endoplasmic reticulum and plays a role in facilitating the assembly of multimeric protein complexes inside the endoplasmic reticulum. Ribosomal proteins, including L22, EF-Ts, were also upregulated only upon recovery to heat stress.

Thirty-six proteins were downregulated only after recovery to heat stress (Additional file 5). Eight downregulated proteins were involved in photosynthesis, including PsbE, PsaD, PetC, PetD, FNR in light reaction and phosphoribulokinase, FBA, fructose-1,6- bisphosphatase in Calvin cycle. Two isoforms of FBA, glyceraldehyde-3-phosphate dehydrogenase and phosphoglycerate kinase involved in glycolysis were also repressed after recovery from heat stress. Down-expressed proteins involved in amino acid metabolism included aspartate aminotransferase, serine-pyruvate aminotransferase, ketol-acid reductoisomerase, and aminomethyltransferase. Catalase (CAT) and APX involved in H_2_O_2_ detoxification were also downregulated after recovery from heat stress. Several proteins from this group were unfortunately unidentified.

## Discussion

One of the many locations for heat stress injury in cells is the membrane. TBARS is the product of lipid peroxidation in plants. The chlorophyll *a* fluorescence transient analysis (O-J-I-P test) is a powerful tool to probe the PSII reactions, which may help determine the state of the electron transport chain [54]. In this study, we investigated the TBARS content and chlorophyll fluorescence parameters in grape leaves under heat stress and subsequent recovery (Figures 1 and 2). These results showed that young grapevines of the 'Cabernet Sauvignon’ varietal were damaged under heat stress at 43°C for 6 h, but they subsequently recovered at 25°C for 18 h. Differential proteomic analysis of grapevines under these two conditions were also performed, and the findings are further discussed below.

### Electron transport chain and related proteins involved in the photosynthesis

**Figure 6:**
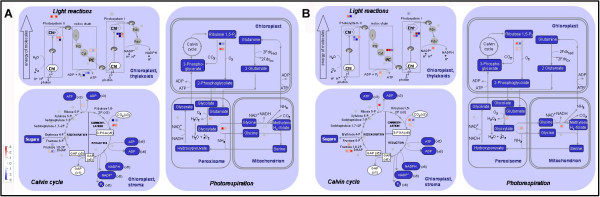
MapMan visualization of photosynthesis in grapevine leaves under heat stress (A) and subsequent recovery (B).

Photosynthesis is known to be one of the most heat sensitive processes due to its complex mechanisms and requirement for enzymes. It is directly related to plant productivity and energy utilization. In this study we identified 34 dysregulated proteins involved in photosynthesis, upon heat stress and subsequent recovery. These accounted for one fifth of all differentially expressed proteins in this study (Table 1 and Figure 6). Moreover, enrichment analysis showed that photosynthesis was enriched under heat stress and/or recovery (Additional file 6 and Additional file 7).

**Table 1:** Proteins involved in photosynthesis under heat stress and/or subsequent recovery

**Protein accession**	**Fold change**		**Bin**	**Species**	**Description**
	**HS**	**RC**			
**A5ASG6**	0.924	2.708	1.1.1.1	*Arabidopsis thaliana*	Photosystem II light harvesting complex protein 2.1, LHCB2.1
**A5BPB2**	0.438	0.524	1.1.1.1	*Arabidopsis thaliana*	Putative light-harvesting chlorophyll-protein complex II subunit B1, LHCB1.4
**A5B5I4**	0.456	1.084	1.1.1.2	*Arabidopsis thaliana*	Chlorophyll *a/b*-binding protein 1, chloroplastic, LHCB1.3
**D7UA58**	0.59	1.176	1.1.1.2	*Gossypium hirsutum*	PsbP precursor
**Q67H94**	1.045	0.608	1.1.1.2	*Muscari comosum*	Cytochrome *b*_559_ subunit alpha (Fragment), PsbE (cytb559α)
**E0CR63**	1.041	1.603	1.1.1.2	*Ricinus communis*	Photosystem II 22 kDa protein, PsbS, chloroplast precursor
**B6VJV1**	0.601	1.928	1.1.1.2	*Vitis vinifera*	Photosystem II protein D1, PsbA (D1)
**A5AWT3**	7.737	2.387	1.1.1.2	*Nicotiana tabacum*	Photosystem II 10 kDa polypeptide, PsbR, chloroplastic
**F6GY64**	NA*	1.645	1.1.1.2	*Populus trichocarpa*	One helix protein 2
**A5AW35**	0.656	1.226	1.1.2.2	*Ricinus communis*	Photosystem I reaction center subunit XI, PsaL, chloroplastic
**A5B2H3**	7.317	1.234	1.1.2.2	*Ricinus communis*	Photosystem I reaction center subunit III, chloroplast precursor, PsaF
**A5AEB4**	0.878	0.582	1.1.2.2	*Ricinus communis*	Photosystem I reaction center subunit II, PsaD, chloroplast precursor
**Q0ZJ20**	0.545	5.057	1.1.2.2	*Vitis vinifera*	photosystem I P700 apoprotein A1, PsaA
**F6I0D9**	28.065	0.185	1.1.2.2	*Medicago truncatula*	Photosystem I reaction center subunit N, PsaN
**A5BHE6**	5.11	2.172	1.1.2.2	*Ricinus communis*	Photosystem I reaction center subunit VI, PsaH
**A5BX41**	0.846	0.236	1.1.3	*Vitis vinifera*	Cytochrome *b*_6_/*f* complex iron-sulfur subunit, PetC
**Q0ZIY8**	1.479	0.417	1.1.3	*Vitis vinifera*	Cytochrome *b*_6_/*f* complex subunit IV, PetD
**Q0ZIY9**	0.8	1.881	1.1.3	*Vitis vinifera*	Cytochrome *b*_6_, PetB
**Q67H40**	0.392	0.818	1.1.4	*Muscari comosum*	ATP synthase subunit beta, chloroplastic
**Q0ZJ34**	8.386	0.957	1.1.4	*Vitis vinifera*	ATP synthase CF (0) b subunit
**F6H7M1**	1.502	1	1.1.4	*Vitis vinifera*	ATP synthase gamma chain, chloroplastic-like isoform 1
**F6HVW3**	1.995	1.03	1.1.4	*Nicotiana tabacum*	ATP synthase delta chain, chloroplastic
**Q95FU2**	1.83	0.401	1.1.4	*Coccoloba uvifera*	ATP synthase beta subunit
**E0CQ75**	1.234	0.554	1.1.5.3	*Ricinus communis*	Ferredoxin--NADP reductase, FNR
**D7TQZ8**	0.666	0.726	1.2.2	*Glycine max*	Peroxisomal (S)-2-hydroxy-acid oxidase GLO1-like
**A5BTM9**	2.969	0.505	1.3.1	*Vitis vinifera*	Ribulose-1,5-bisphosphate carboxylase/oxygenase large subunit, RbcL
**Q2I314**	1.627	1.886	1.3.2	*Vitis pseudoreticulata*	ribulose-1,5-bisphophate carboxylase/oxygenase small subunit
**A5BHS5**	0.61	1.167	1.3.4	*Glycine max*	NADP-dependent glyceraldehyde-3-phosphate dehydrogenase-like
**F6HFL6**	1.833	0.733	1.3.6	*Vitis vinifera*	Fructose-bisphosphate aldolase, FBA
**F6GWQ0**	0.799	0.626	1.3.6	*Vitis vinifera*	Fructose-bisphosphate aldolase
**A5AYR7**	1.476	0.664	1.3.7	*Glycine max*	Fructose-1,6-bisphosphatase, chloroplastic-like
**A5BE19**	0.84	0.447	1.3.12	*Vitis vinifera*	Phosphoribulose kinase, putative
**D7THJ7**	0.482	0.739	1.3.13	*Ricinus communis*	Ribulose bisphosphate carboxylase/oxygenase activase 1, chloroplast precursor
**F6HBT1**	0.594	1.004	1.3.13	*Glycine max*	Ribulose bisphosphate carboxylase/oxygenase activase, chloroplastic-like

*The proteins were not quantified under heat stress or subsequent recovery.

HS refers to the fold change in heat stressed proteins, with respect to controls, while RC refers to the fold change in proteins after recovery, with respect to controls.

PSII is thermally labile and is considered to be the most sensitive component of the electron transport chain [55,56]. The peripheral antennas of PSII are composed of major trimeric and minor monomeric LHCII proteins. In this study, the expression of LHCII1.3 and LHCII1.4 was inhibited under heat stress and increased after recovery, which indicated that LHCII1.3 and LHCII1.4, might be thermally labile. LHCB2.1 showed the same expression as control under heat stress while increased about 2.7 fold after recovery, suggesting that LHCB2.1 may be thermostable and solely involved in the recovery from heat stress. The OEC activity is in close association with the 33 kDa (PsbO) and 23 kDa (PsbP). PsbO is a key structural component of many different types of OECs and functions to stabilize the manganese cluster and modulate the Ca^2+^ and Cl^-^ requirements for oxygen evolution [57]. Additionally, the 10-kDa PsbR protein has also been found play a role in stable association of the PsbP with the PSII core for water oxidation [57,58]. In the present study, PsbO-2 levels were not altered upon heat stress or subsequent recovery, the PsbP precursor was repressed under heat stress but returned to control level after subsequent recovery, while PsbR was elevated approximately eight fold with respect to its control under heat stress, and remained upregulated two fold upon subsequent recovery. In addition, the chlorophyll fluorescence parameter W_k_ showed that the OEC of PSII was damaged under heat stress, but returned back to the normal physiological level in the recovery phase (Figure 2). Therefore, these combined results suggest that PsbR may play an important role in maintaining the stability of the OEC of PSII compared to PsbO and PsbP in grape leaves.

In the present study, RC_QA_ values decreased under heat stress and increased to the control level after subsequent recovery (Figure 2), indicating that the PSII reaction center was inhibited by heat stress and then recovered when the stress was removed. The change of D1 protein corroborated this result (Table 1). The multi-subunits (PetA, PetB, PetC and PetD) complex of Cyt*b*_*6*_/*f* is a crucial component for the acceptor side of electron transport chain of PSII [59]. In the present study, three subunits PetB, PetD and PetC were differentially expressed. The expression level of PetB, PetC and PetD did not change significantly under heat stress, however, after recovery, the expression of PetC and PetD was largely inhibited while PetB was induced. In addition, *φ*_Eo_ and *ψ*_Eo_ were reduced in grape leaves under heat stress, then returned to control levels with the subsequent recovery (Figure 2). This suggests that the function of the acceptor portion of the electron transport chain of PSII including Cyt*b*_*6*_/*f* complex recovered from heat stress. These combined results suggest that PetB may promote the Cyt*b*_*6*_/*f* complex to recover from heat stress.

The study showed that many proteins in the PSI complex changed upon heat stress (Table 1). PSI consists of a core complex and a peripheral antenna. In plants, these two functional units result from the assembly of at least 19 protein subunits. The PSI core complex contains 15 subunits, including PsaA to PsaL and PsaN to PsaP which play important roles in PSI function. For example, PsaF is located in the thylakoid lumen, and contains a lysine-rich helix-loop-helix motif that has been demonstrated to interact with plastocyanin in plants and with plastocyanin (PC) or Cytochrome *c*_*6*_ in algae [60]. PsaN is necessary for the docking PC to the PSI complex, and is the only subunit located entirely on the lumenal side of PSI. In the present study, it was shown from the chlorophyll *a* fluorescence parameter *δ*_Ro_ that PSI was damaged under heat stress and recovered to the control level when returned to normal temperatures (Figure 2). Consistent with this observation, the levels of PsaA and PsaL declined under heat stress. However, the expression level of PsaA remained 5 fold higher compared to the control after subsequent recovery, suggesting that PsaA may have a positive effect in the recovery phase of PSI. In addition, the expression of PsaF, PsaH and PsaN increased by a 7.3, 5.1 and 28.1 fold respectively under heat stress, which indicated that PsaF, PsaH and PsaN might play a role of protection from heat stress in the PSI complex of grape leaves. It is especially interesting that while all proteins of the PSI complex inhibited under heat stress were hydrophobic, all proteins induced under heat stress were hydrophilic.

ATP synthase produces ATP from ADP in the presence of a proton gradient across the membrane. F-type ATPase has two components, CF (1) - the catalytic core - and CF (0) - the membrane proton channel. CF (1) has five subunits: α, β, γ, δ and ϵ while CF (0) has four main subunits: a, b, b′ and c. The α chain is the largest subunit of the ATP synthase. The γ chain is believed to be important in regulating ATPase activity and the flow of protons through the CF (0) complex. In the study, all the identified ATP synthase subunits (γ, δ and b of CF (0)) were upregulated under heat stress, and all of them recovered to their control levels after subsequent recovery. Especially, the expression of subunit b is increased by 8.4 fold under heat stress. These result suggested that these three subunits may have a protective role against heat stress for ATP synthase, and continue to provide energy for maintaining the normal physiological processes of grapevines.

### Proteins involved in abiotic stress and redox regulation

Nineteen identified dysregulated proteins were functionally characterized as being involved in stress response (Table 2). Most of them were assigned to one of the four major classes of molecular chaperones, HSP90, HSP70, HSP60 and sHSPs, however, no proteins belonged to HSP100 family. Plants respond to different abiotic stress by inducing the synthesis of proteins from the heat shock protein (HSP)/chaperone family which have been shown to play a crucial role in protecting plants against stress by re-establishing normal protein conformations and thus cellular homeostasis [61]. In this study, nine HSPs were differentially expressed under heat stress or after subsequent recovery. Proteins from the HSP90 family do not only manage protein folding [62,63], but also play a major role in signal-transduction networks, cell-cycle control, protein degradation and protein trafficking [64-66]. A previous study in *P. euphratica* showed that a putative HSP90 was upregulated early upon heat stress and later returned to control values [14]. In our study, three members of HSP90 family were identified and differentially expressed. Two of them were inhibited, while the expression of HSP90-1 was not affected by heat stress. However, all of them were upregulated during subsequent recovery. Proteins from the HSP70 family are essential for preventing aggregation and assisting re-folding of non-native proteins under both normal and stressing environmental conditions [62,67]. They are involved in protein import and translocation processes, and in facilitating the proteolytic degradation of unstable proteins by targeting these proteins to lysosomes or proteasomes [62]. Previous reports have documented that HSP70 were accumulated under heat stress [9,68] . In our research, three members of the HSP70 family were identified. One of the HSP70 family proteins was repressed under heat stress and recovered to the control level during the subsequent recovery (Table 2) while the other two had no difference compared to their control under heat stress but were downregulated during the recovery phase (Table 2). This suggests that the many isoforms of HSP70 play different roles under heat stress. In plants, the sHSPs are abundant and diverse, and can be classified into five families according to their cellular localization; including cytosol (class I and II), chloroplast (class III), endoplasmic reticulum (class IV), and mitochondrion (class V) [9,69-71]. In addition, sHSPs have been reported to be involved in protecting macromolecules like enzymes, lipids, nucleic acid, and mRNAs from dehydration [72]. In our study, one protein (HSP22) was predicted to be an endoplasmic reticulum-targeted sHSP, whereas the other sHSP (HSP21) was predicted to be chloroplast-targeted. A previous study in Arabidopsis showed the expression of HSP21 and HSP22 significantly increased under heat stress [73]. In our study, the similar results were observed, and moreover, the expression of HSP21 and HSP22 return to control levels after subsequent recovery. This also agrees with our previous findings, in which the mRNA level of HSP21 and HSP22 exhibited similar increases [29]. In addition, increased thermotolerance has been previously achieved by overexpressing the plastidial Hsp21 in tomato [74]. Therefore, these sHSPs may have the important functions in alleviating heat stress in grapevines.

**Table 2:** Proteins involved in abiotic stress and redox under heat stress and/or subsequent recovery

**Protein accession**	**Fold change**		**Bin**	**Species**	**Description**
	**HS**	**RC**			
**A5BS35**	0.412	1.031	20.1	*Nicotiana tabacum*	NtPRp27
**A5C2C9**	0.877	0.324	20.1	*Ricinus communis*	Protein MLO, putative
**A5AHJ5**	0.114	1.149	20.2	*Vitis vinifera*	Osmotin-like protein
**F6HYG1**	0.409	0.933	20.2.1	*Ricinus communis*	Heat shock 70 kDa protein
**F6HJZ4**	3.046	0.978	20.2.1	*Corylus heterophylla*	Heat shock protein 22, endoplasmic reticulum, HSP22
**A5B868**	5.531	1.45	20.2.1	*Solanum lycopersicum*	Heat shock protein 21, chloroplast, HSP21
**F6HU55**	0.878	1.989	20.2.1	*Cucumis sativus*	Heat shock protein 70
**F6HYK6**	1.005	2.591	20.2.1	*Vitis vinifera*	Similar to PsHSP71.2
**A5ADL7**	1.312	2.959	20.2.1	*Arabidopsis thaliana*	Heat shock protein 90.1, cytoplasmic, HSP90-1
**A5BX00**	0.382	1.782	20.2.1	*Arabidopsis thaliana*	HSP90-like protein 7, HSP90-7
**F6HGF1**	0.598	2.854	20.2.1	*Ipomoea nil*	Heat shock protein 90
**E0CVB4**	3.416	1.983	20.2.1	*Nicotiana tabacum*	Heat shock protein 26
**F6HKZ7**	5.463	0.748	20.2.99	*Ricinus communis*	ATOZI1
**D5LN28**	1.8	0.472	20.2.99	*Vitis pseudoreticulata*	Universal stress protein (USP) family protein
**E0CQM3**	9.846	2.943	21.1	*Populus trichocarpa*	Thioredoxin M
**D7SKR5**	1.32	0.648	21.2.1	*Vitis vinifera*	Ascorbate peroxidase, APX
**F6H0K6**	1.508	0.434	21.2.1	*Glycine max*	L-ascorbate peroxidase T, chloroplastic-like isoform 2
**F6HTX9**	4.904	0.99	21.6	*Vitis vinifera*	Cytoplasmic [Cu-Zn] SOD
**D7UD99**	1.071	0.604	21.6	*Vitis vinifera*	Catalase, CAT

**Figure 7:**
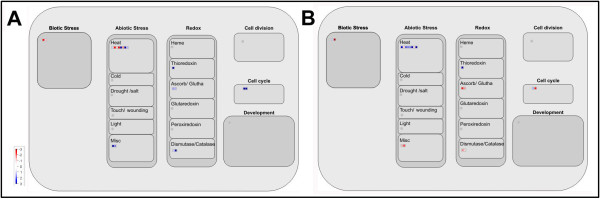
Overview of cellular response in grapevine leaves under heat stress (A) and subsequent recovery (B) visualized by MapMan.

The antioxidant enzymes are known to play important roles in scavenging or reducing excessive reactive oxygen species (ROS) which are produced under stress conditions, in order to maintain cell redox homeostasis [9]. In this study, we identified a group of antioxidant enzymes including [Cu-Zn] SOD, CAT, APX and thioredoxin. [Cu-Zn] SOD which plays a central role in protecting against oxidative stress is generally found in the cytosol and chloroplasts (Table 2). The cytoplasmic [Cu-Zn] SOD showed considerable upregulation (approximately 5 fold) under heat stress, followed by a return to the control level after subsequent recovery. This is in agreement with published results in the heat-tolerant *Agrostis scabra*, while these redox proteins were not detected in the heat-sensitive *Agrostis stolonifera*[75]. In addition, the expression of APX increased under heat stress in our study. Thioredoxins are small proteins catalyzing thiol-disulfide interchange, which is involved in the regulation of the redox environment in cells [76,77]. The most prominent candidates of proteins are thioredoxin h in *Populus euphratica* Oliv. and rice leaves, upon heat stress [9,14]. Thioredoxin M4 was predicted to be located in chloroplast in our study, and was upregulated almost 10 fold under heat stress and maintained approximately 3 fold after subsequent recovery (Table 2). These results suggest that cytosolic [Cu-Zn] SOD, APX and chloroplastic thioredoxin have important roles in maintaining redox homeostasis in grapevine cells under heat stress (Figure 7).

### Proteins involved in metabolism

The expression of most proteins predicated to be involved in metabolism was slightly downregulated in grape leaves under heat stress (Table 3), indicating that the metabolism of 'Cabernet Sauvignon’ grapevine was mildly affected under heat stress. In the present study, three proteins identified were involved in nucleotide metabolism. Most significantly, NDPK1, which plays a major role in the synthesis of nucleoside triphosphates other than ATP was upregulated more than 10 fold under heat stress, and declined to 1.7 fold following recovery, compared to controls. Fukamatsu *et al.* showed that Arabidopsis NDPK1 is a component of ROS signaling pathways by interacting with three CATs [78]. Furthermore, in *Neurospora crassa*, NDPK1 is suggested to control CATs in response to heat, oxidative stress and light, and results have indicated that NDPK1 protein was translocated from the plasma membrane to the cytoplasm in response to light, and may interact with CAT [79]. Together with our findings, we suggest that NDPK1 may play an important role in grape leaves in response to heat stress.

**Table 3:** Proteins involved in metabolism under heat stress and/or subsequent recovery

**Protein accession**	**Fold change**		**Bin**	**Species**	**Description**
	**HS**	**RC**			
**D7TDB6**	0.547	1.053	2.1.2.1	*Vitis vinifera*	ADP-glucose pyrophosphorylase catalytic subunit
**F6HDM4**	0.516	1.178	2.1.2.1	*Vitis vinifera*	Glucose-1-phosphate adenylyltransferase
**Q9S944**	0.169	0.882	2.2.1.3.3	*Vitis vinifera*	Vacuolar invertase 1, GIN1
**F6HJU7**	0.667	1.854	3.1.2.2	*Ricinus communis*	Stachyose synthase precursor
**E0CU00**	0.805	0.542	3.5	*Ricinus communis*	Aldo/keto reductase
**F6HHH7**	NA*	1.714	3.5	*Glycine max*	Putative aryl-alcohol dehydrogenase C977.14c-like
**D7TMQ2**	0.666	0.783	6.1	*Vitis vinifera*	Citrate synthase, glyoxysomal
**F6HJJ4**	0.624	1.095	6.3	*Ricinus communis*	Malate dehydrogenase
**A5BEJ8**	0.524	1.328	6.3	*Vitis vinifera*	Malate dehydrogenase, putative
**F6H9P9**	0.455	1.618	11.1.1	*Camellia oleifera*	Biotin carboxylase, CAC2
**G3G8J7**	0.425	0.696	12.1.2	*Vitis vinifera*	Nitrite reductase 1
**D7SW04**	0.744	0.519	13.1.1.2.1	*Petunia x hybrida*	Prephenate aminotransferase
**A5ACX0**	0.389	1.125	13.1.1.3.1	*Arabidopsis thaliana*	Alanine-2-oxoglutarate aminotransferase 2
**F6HA09**	0.696	0.616	13.1.1.3.11	*Ricinus communis*	Serine-pyruvate aminotransferase
**F6GST3**	0.614	0.949	13.1.2.3.22	*Ricinus communis*	Argininosuccinate synthase
**A5AGN5**	0.896	0.157	13.1.4.1	*Catharanthus roseus*	Ketol-acid reductoisomerase
**A5AFH5**	0.397	0.825	13.1.5.3.1	*Vitis vinifera*	Cysteine synthase
**F6HHQ7**	1.652	0.553	13.2.3.5	*Hevea brasiliensis*	Acetyl-CoA C-acetyltransferase
**F6H7I9**	1.038	0.559	13.2.5.2	*Vitis vinifera*	Aminomethyltransferase, mitochondrial-like
**A5BQ64**	1.09	0.474	16.1.3.3	*Hevea brasiliensis*	2-methyl-6-phytylbenzoquinone methyltranferase
**A5BJL8**	0.5	0.376	16.4.3.1	*Vitis vinifera*	Cyanate hydratase
**D7SLA9**	0.835	0.627	17.7.1.2	*Vitis vinifera*	Lipoxygenase
**A5BEM6**	1.25	0.643	19.3	*Ricinus communis*	Glutamate-1-semialdehyde-2,1-aminomutase, GSA-AT
**A5BF85**	1.526	0.635	19.8	*Ricinus communis*	Coproporphyrinogen III oxidase, CPOX
**F6HM73**	0.353	0.358	19.10	*Ricinus communis*	Magnesium-chelatase subunit chlI, chloroplast precursor
**F6HL38**	0.2	1.02	23.3.1.3	*Glycine max*	Uracil phosphoribosyltransferase-like
**A5B878**	10.227	1.695	23.4.10	*Vitis vinifera*	Nucleoside diphosphate kinase 1, NDPK1
**F6HBJ7**	0.813	0.602	23.4.99	*Ricinus communis*	Inorganic pyrophosphatase

*The proteins were not quantified under heat stress or subsequent recovery.

### Proteins involved in glycolysis and TCA in mitochondrial respiration

The regulation of the enzymes involved in respiratory carbon metabolism under heat stress has been a subject of debate. As shown in Table 4, there were six enzymes identified that are involved in glycolysis, which did not significantly change in expression level under heat stress while were downregulated after subsequent recovery. In addition, we found that five enzymes (dihydrolipoyl dehydrogenase, aconitase, malate dehydrogenase, succinate-semialdehyde dehydrogenase and carbonic anhydrase), which are involved in the TCA cycle, were dysregulated in the study. With the exception of aconitase, the expression of these enzymes was inhibited under heat stress and recovered to the control level or showed a slight increase after subsequent recovery. The above results suggest that the glycolysis pathway was not influenced, while the TCA cycle was inhibited by heat stress. We also hypothesize that during recovery, the TCA cycle recovereds to control levels to consume the excess pyruvic acid produced by glycolysis. Therefore, the glycolysis pathway may be more heat tolerant than the TCA cycle in respiration in grapevines.

**Table 4:** Proteins involved in respiration under heat stress and subsequent recovery

**Protein accession**	**Fold change**		**Bin**	**Species**	**Description**
	**HS**	**RC**			
**F6I0H8**	0.548	1.144	4.1	*Gossypium hirsutum*	UDP-D-glucose pyrophosphorylase
**F6HFF7**	0.931	1.93	4.2	*Ricinus communis*	Phosphoglucomutase
**A5B118**	1.356	0.481	4.7	*Vitis vinifera*	Fructose-bisphosphate aldolase, FBA
**A5BX43**	1.222	0.461	4.7	*Vitis vinifera*	Fructose-bisphosphate aldolase, FBA, cytoplasmic isozyme 1-like
**F6GSG7**	1.105	0.653	4.9	*Ricinus communis*	Glyceraldehyde 3-phosphate dehydrogenase
**A5CAF6**	1.017	0.5	4.10	*Vitis vinifera*	Phosphoglycerate kinase, cytosolic-like
**A5BGC9**	0.507	1.296	7.1.3	*Vitis vinifera*	6-phosphogluconate dehydrogenase
**A5BDU8**	0.537	0.938	8.1.1.3	*Vitis vinifera*	Dihydrolipoamide dehydrogenase, putative
**D7TEL2**	0.672	1.64	8.1.3	*Ricinus communis*	Aconitase
**F6HZK0**	0.499	1	8.2.9	*Vitis vinifera*	Malate dehydrogenase
**F6H9T6**	0.466	0.972	8.2.99	*Solanum lycopersicum*	Succinic semialdehyde dehydrogenase
**A5BQL5**	0.628	0.795	8.3	*Vitis vinifera*	Chloroplast carbonic anhydrase
**A5C9C0**	0.833	1.722	9.1.2	*Ricinus communis*	NADH-ubiquinone oxidoreductase flavoprotein
**A5ASP2**	1.286	2.886	9.1.2	*Ricinus communis*	NADH-ubiquinone oxidoreductase 24 kD subunit
**D7TQ15**	NA*	2.667	9.1.2	*Solanum tuberosum*	NADH:ubiquinone oxidoreductase-like
**D7SUP9**	NA*	0.653	9.5	*Camellia sinensis*	Ubiquinol-cytochrome C reductase complex

*The proteins were not quantified under heat stress or subsequent recovery.

## Conclusion

This study provides a global look at the dysregulated proteins in grapevine leaves exposed to heat stress and after subsequent recovery using the iTRAQ technique. A total of 174 differentially expressed proteins were identified in response to heat stress and/or subsequent recovery. On the basis of these findings, we propose that some proteins related to the electron transport chain of photosynthesis, antioxidant enzymes, HSPs and the glycolysis pathway may play key roles in protecting grapevines from heat stress and enhancing their recovery capacity.

## Methods

### Plant materials and treatments

One-year old 'Cabernet sauvignon’ (*V. vinifera* L*.*) grapevine cuttings were planted in pots, then grown in a greenhouse at 70-80% relative humidity under a 18-25°C, with the maximum photosynthetically active radiation (PAR) at approximately 1,000 μmol photons m^-2^ s^-1^. When the sixth leaves (from bottom to top) of grapevines became mature, all grapevines were divided into two groups and acclimated for two days in a controlled environment room (70% average relative humidity, 25/18 (12 h/12 h) day/night cycle and PAR at 800 μmol m^-2^ s^-1^). On day three, the grapevines were subjected to the following treatments: (1) the plants of the control group were maintained at the optimal day/night temperature (25°C/18°C) in the above growth chamber; (2) the plants of the treatment group were exposed to 43°C from 9:30 to 15:30 (the conditions were the same as the control, except for temperature). The stressed grapevines were then allowed to recover at 25°C rapidly (from 43°C to 25°C in about 10 min), then, all conditions were the same as the control until 9:30 h on Day 4. The fourth to sixth leaves (from bottom to up) of each plant were detached from each plant at 15:30 Day 3 (the end of the heat stress treatment) and 9:30 Day 4 (the day of recovery) (Additional file 8). Each biological replicate included three plants, and three replicates were used for both treatment and controls. Leaves were frozen in liquid nitrogen immediately and stored at -80°C for further analysis.

### Analysis of chlorophyll fluorescence parameters

The chlorophyll *a* fluorescence transient (O-J-I-P test) was measured by a Handy Plant Efficiency Analyzer after the leaves adapted for 15 min in the dark. The chlorophyll *a* fluorescence transient was induced by a saturating photon flux density at 3000 μmol photons m^-2^ s^-1^, provided by an array of six light-emitting diodes (peak 650 nm). The fluorescence signals were recorded within a time span from 10 μs to 1 s, with a data acquisition rate of 10 μs for the first 2 ms and every 1ms thereafter. The following data from the original measurements were used: *F*_k_: fluorescence intensity at 300 μs [required for calculation of the initial slope (M) of the relative variable fluorescence (V) kinetics and W_k_]; *F*_j_: the fluorescence intensity at 2 ms (the J-step); *F*_i_: the fluorescence intensity at 30 ms (the I-step); *F*_m_: maximal fluorescence intensity (the P-step). The derived parameters are as follows: *F*_o_: fluorescence intensity at 50 μs; the parameter W_k_ on donor side of photosystem II (PSII), represents the damage to OEC, W_k_ = (*F*_k_-*F*_o_)/(*F*_j_-*F*_o_); the parameter RC_QA_ on reaction center of PSII, represents the density of Q_A_-reducing reaction centers, RC_QA_ = φ_Po_ × (V_j_/M_o_) × (ABS/CS_m_); the parameter *F*_v_/*F*_m_ on acceptor side of PSII, represents maximum quantum yield of primary photochemistry at t = 0; the parameter φ_Eo_ on acceptor side of PSII, represents quantum yield for electron transport (at t = 0), φ_Eo_ = ET_o_/ABS = (*F*_m_-*F*_j_)/*F*_m_. The calculation and derivation of a range of new parameters from O-J-I-P transients is shown in Additional file 9. Five independent replicates were used in both treatments and controls respectively, and each replicate consisted of a plant. The chlorophyll *a* fluorescence transient was measured on the same plants under heat stress and subsequent recovery.

### Measurement of thiobarbituric acidreactivesubstances (TBARS)

The content of TBARS was determined according to the methods of Heath and Packer [80] with minor modifications. About 1 g of frozen leaves were homogenized in 0.5% thiobarbituric acid and 20% trichloroacetic acid. After heating for 30 min at 95°C, samples were cooled quickly in an ice-water bath. Air bubbles were then removed from each tube by shaking, and samples were centrifuged at 14,000 rpm for 20 minutes at 20°C. The absorbance of the supernatant was read at 532 nm, corrected for nonspecific turbidity by subtracting the absorbance at 600 nm. The amount of TBARS was calculated by using an extinction coefficient of 155 mM^-1^ cm^-1^.

### Protein extraction

Total protein was extracted using the cold-acetone method. The three biological replicates of the frozen grape leaves were pooled for iTRAQ analysis [81,82], and 10% m/m polyvinyl polypyrrolidone (PVPP) were transferred to a mortar with liquid nitrogen and ground until a fine powder was obtained. Approximately 500 mg of the ground up leaf powder was combined with 4 ml of 10% m/v trichloroacetic acid (TCA) in acetone to each sample, and the samples were incubated at -20°C for 2 h. The samples were then centrifuged at 20,000 *g* for 30 min at 4°C. The supernatant was discarded without disturbing the pellets. In order to reduce acidity, the pellets were washed with acetone and incubated at -20°C for 30 min, and centrifuged at 20,000 *g* for 30 min at 4°C. The washing step with acetone was repeated several times until the pellets were white. The dried pellets were lysed with 1 ml protein extraction reagent [8 M urea, 30 mM HEPES, 1 mM PMSF, 2 mM EDTA and 10 mM DTT]. The pellets were then dissolved by ultrasound (pulse on 2 s, pulse off 3 s, power 180 w) for five minutes. After dissolution, the solution was centrifuged at 20,000 *g* for 30 min at 4°C to remove non-soluble impurities. Proteins were reduced with 10 mM DTT at 56°C for 1 h and alkylated immediately by 55 mM iodoacetamide (IAM) in the dark at room temperature for 1 h. The treated proteins were precipitated in acetone at -20°C for 3 h. After centrifugation at 20,000 *g* for 20 min at 4°C, the pellets were resuspended and ultrasonicated in pre-chilled 50% TEAB buffer with 0.1% SDS and dissolved by ultrasound. The proteins were regained after centrifugation at 2000 *g* and protein concentration was determined by the Bradford assay using BSA as a standard.

### Digestion and iTRAQ labeling

Total of 100 μg protein in TEAB buffer was incubated with 3.3 μg of trypsin (1 μg/μl) (Promega, Madison, WI, USA) at 37°C for 24 h in a sealed tube. The tryptic peptides were lyophilized and dissolved in the 50% TEAB buffer and the trypsin digested samples were analyzed using MALDI-TOF/TOF to ensure complete digestion. The protocol of iTRAQ labelling was followed the company manual. The tryptic peptides were incubated with 8-plex iTRAQ labeling kit (AB Sciex, Foster City, CA, USA) (116 for HS-CK; 121 for HS-TR; 114 for RC-CK; 118 for RC-TR) for 2 h at room temperature, which was dissolved in 70 μl isopropanol.

### Peptide fractionation by strong cation exchange (SCX)

The labeled samples were fractionated using an HPLC system (Shimadzu, Kyoto, Japan) connected to an SCX column (Luna 5u column, 4.6 mm × 250 mm, 5 μm, 100 Å; Phenomenex, Torrence, CA). The retained peptides were eluted using Buffer A (10 mM KH_2_PO_4_ in an aqueous solution of 25% acetonitrile and acidified to a pH of 3.0 with H_3_PO_4_) and Buffer B, where Buffer B was composed of Buffer A with 2 M KCl. The fractions were collected in 1.5 ml microfuge tubes with flow rate at 1 ml/min. The following chromatographic gradient was applied: 0 ~ 25 min 100% Buffer A; 25 ~ 26 min 5% Buffer B; 26 ~ 46 min 5-30% Buffer B; 46 ~ 51 min 30-50% Buffer B, 51-56 min 50% Buffer B; 56–61 min increasing to 100% Buffer B. All solutions used were centrifuged again at 20,000 *g* for 30 min at 4°C. Fraction collection started 26 min after the injection with a sample collected every 1 min to obtain a total of 38 fractions. For fractions containing a high concentration of salt, an additional step was used to remove the salt with Strata-X 33u polymeric reversed phase column (Phenomenex). Eluted fractions were dried in a vacuum concentrator, and each fraction was dissolved in 0.1% formic acid solution prior to reversed-phase nano-LC-tandem mass spectrometry (LC-MS/MS).

### Reverse-Pphase nano liquid xhromatography tandem MS

The SCX peptide fractions were pooled together to obtain 10 final fractions, to reduce the number of samples and collection time. A 10 μl sample from each fraction was injected twice to the Proxeon Easy Nano-LC system. Peptides were separated on C18 analytical reverse phase column (100 mm × 75 mm, 300 Å, 5 μm) at a flow rate of 400 nl/min and a linear LC gradient profile was used to elute peptides from the column. The fractions were then analyzed using a hybrid Quadrupole/Time-of-flight MS (Triple-TOF 5600, AB SCIEX, USA) with nano electrospray ion source. The MS/MS scans from 50–2000 m/z were recorded. Nitrogen was used as the collision gas. The ionization tip voltage and interface temperature were set at 1250 V and 150°C, respectively.

### Database search and protein quantification

All the mass spectral data were collected using Micro TOF (AB5600, Applied Biosystems) control software, and processed and analyzed using Data Analysis 4.0. The database of *uniprot_grape* (12/1/2011, 55416 sequences) was downloaded (http://www.uniprot.org/) and integrated into the Mascot search engine version 2.3.01 by its database maintenance unit. All parameters were set as follows: specifying trypsin as the digestion enzyme, cysteine carbamido methylation as fixed modification, iTRAQ 8-Plex on N-terminal residue, iTRAQ 8-Plex on tyrosine, iTRAQ 8-Plex on lysine, glutamine as pyroglutamic acid and oxidation on methionine as the variable modification. The tolerance settings for peptide identification in Mascot searches were set at 0.05 Da for MS and 0.05 Da for MS/MS. The maximum missed cleavages were set as 1. Finally, the Mascot search results were exported into a DAT file, quantified using Mascot 2.3.01 with the following criterias: protein ratio type = median, minimum unique peptides = 1, peptide threshold type = at least homolog. Peptides were not quantified for the following reasons: peptide score was too low, or the deviation was too large. The final ratios of protein were then normalized by taking the median of all the proteins quantified. All quantified proteins are listed in Additional file 10.

### Functional classification, enrichment analysis and subcellular localization

Differentially expressed proteins functionally classified according to MapMan ontology [83]. Enrichment analysis was conducted using the Singular Enrichment Analysis (SEA) tool in the agriGO toolkit [84]. Uniprot IDs were submitted to the SEA tool as the query list and suggested backgrounds were as the select reference. Under advanced options the statistical test method chosen was Fisher, the multi-test method was Yekutieli (FDR under dependency), the significance level was 0.05, and the gene ontology type chosen was Plant GO slim. Subcellular localizations of proteins were determined using TargetP [85].

## Abbreviations

APX: Ascorbate peroxidase; CAT: Catalase; CK: Control; FBA: Fructose bisphosphate aldolase; HS: Heat stress; HSP: Heat shock protein; iTRAQ: Isobaric tags for relative and absolute quantitation; LHC: Light-harvesting chlorophyll-protein complex; NDPK: Nucleoside diphosphate kinase; OEC: Oxygen evolution complex; PS: Photosystem; RC: Recovery; SOD: Superoxide dismutase; TBARS: Thiobarbituric acidreactivesubstances; TCA: Tricarboxylic acid cycle; TR: Treatment.

## Competing interests

The authors declare that they have no competing interests.

## Authors’ contributions

GTL and LJW designed the study, performed the proteomic experiments and wrote the manuscript. LM assisted with experiment design, proteomic experiments, data analysis and manuscript writing. WD, JHL, HGX and BFY assisted to conduct the measurement of TBARS and chlorophyll fluorescence parameters. XQY performed the blast analysis and wrote this part. BCW and SHL revised the draft of the manuscript. All authors read, revised and approved the final manuscript.

## Supplementary Material

Additional file 1 **The homologs of unknown proteins.** BLASTP (http://www.ncbi.nlm.nih.gov/BLAST/) was used to search for homologs of the unknown proteins. Click here for file

Additional file 2 The functional categories of the 174 differentially expressed proteins according to MapMan ontology. Click here for file

Additional file 3 Differentially expressed proteins under heat stress and subsequent recovery. Click here for file

Additional file 4 Differentially expressed proteins only response to heat stress. Click here for file

Additional file 5 Differentially expressed proteins only response to recovery from heat stress. Click here for file

Additional file 6 The temperature conditions of grapevine in the present study. Click here for file

Additional file 7 Enrichment analysis against agriGO of grapevine proteins under heat stress and/or subsequent recovery. Click here for file

Additional file 8 File containing the GO-terms annotated by agriGO for the proteins differentially expressed under heat stress and/or subsequent recovery. Click here for file

Additional file 9 **Summary of parameters, formulae and their description using data extracted from chlorophyll *a *** **fluorescence transient (O-J-I-P test)**. Click here for file

Additional file 10 Detailed information of the identified proteins under heat stress and/or subsequent recovery. Click here for file
