# Supplementary figures and images for: Differential proteomic analysis of grapevine leaves by iTRAQ reveals responses to heat stress and subsequent recovery
Source: BMC Plant Biol. 2014 Apr 28;14:110. doi: 10.1186/1471-2229-14-110 (PMC4108046; doi:10.1186/1471-2229-14-110)

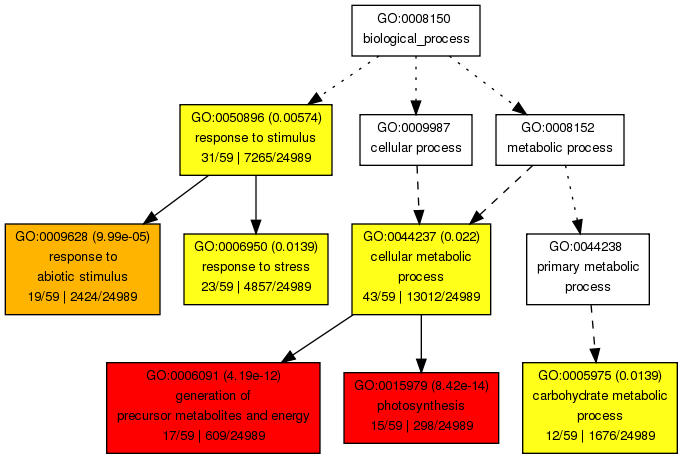

Supplement: Additional file 6 — The temperature conditions of grapevine in the present study. [file 1471-2229-14-110-S6.png]

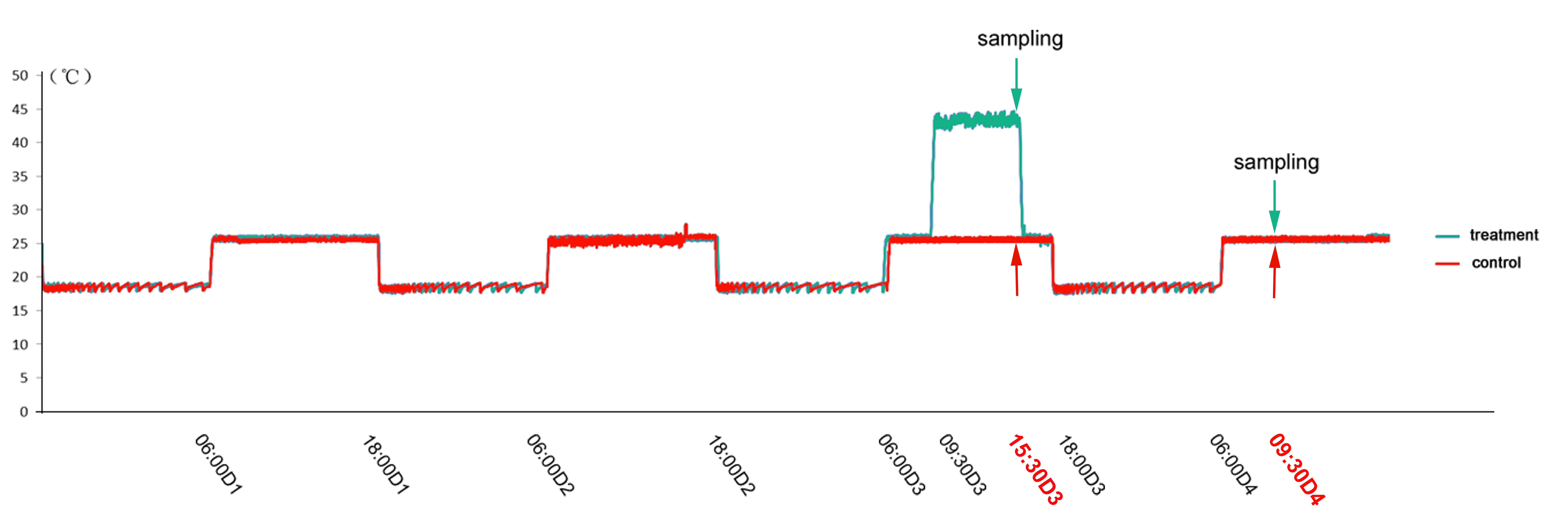

Supplement: Additional file 8 — File containing the GO-terms annotated by agriGO for the proteins differentially expressed under heat stress and/or subsequent recovery. [file 1471-2229-14-110-S8.tiff]
